# The Effects of Milling Conditions on the Particle Size, Quality, and Noodle-Making Performance of Whole-Wheat Flour: A Mortar Mill Study

**DOI:** 10.3390/foods14091609

**Published:** 2025-05-01

**Authors:** Jeonghan Moon, Yujin Moon, Meera Kweon

**Affiliations:** 1 Department of Food Science and Nutrition, Pusan National University, Busan 46241, Republic of Korea; zzx276@pusan.ac.kr (J.M.); amoebacul@pusan.ac.kr (Y.M.); 2 Kimchi Research Institute, Pusan National University, Busan 46241, Republic of Korea

**Keywords:** whole-wheat flour, mortar milling, particle size distribution, starch damage, dough mixing properties, noodle-making performance

## Abstract

In this study, we investigated the effects of mortar milling conditions on the quality and noodle-processing suitability of whole-wheat flours (WWFs). The WWFs were milled at varying pestle speeds (50 and 130 rpm) and for varying durations (10, 20, 40, and 60 min) and analyzed to determine their particle size distribution, physicochemical properties, dough-mixing characteristics, antioxidant activities, and noodle-making performance. High pestle speed (Group H) produced significantly smaller particle sizes, higher flour temperatures, greater moisture loss, and increased starch damage compared to that produced at low pestle speeds (Group L). Compared with Group L, Group H exhibited higher water and sodium carbonate solvent-retention capacity (SRC) values, increased pasting viscosities, and greater gluten strength owing to finer particles. Total phenolic content increased with reduced particle size, whereas antioxidant activity (ABTS radical scavenging) exhibited inconsistent trends. Fresh noodle properties varied with milling conditions; finer WWF particles improved dough resistance but reduced extensibility when water was adjusted according to water SRC. Thus, WWF particle size strongly influences flour functionality and noodle quality, which highlights the need for precise milling control. This study demonstrates, for the first time, the applicability of a mortar-type mill for producing WWFs, with implications for enhancing WWF functionality.

## 1. Introduction

Wheat is one of the most cultivated grains in the world and the second most consumed grain in Korea after rice. Despite annual wheat consumption reaching 38.1 kg per capita, the self-sufficiency rate for domestically produced wheat in Korea is negligible at approximately 1%, leading to considerable imports from the USA, Australia, and Canada [1]. Efforts have been made to develop new wheat varieties through breeding to increase domestic wheat production and consumption. ‘Hojoong’ is a representative semi-soft red winter wheat (*Triticum aestivum* L.) cultivar characterized by low ash content (0.38%), low amylose content (20.5%), and medium protein content (11.3%). These qualities result in a low sodium dodecyl sulfate (SDS) sedimentation volume, high pasting viscosity, and bright flour color, rendering it well suited for noodle production [2].

The demand for whole-wheat flour (WWF) products has increased, owing to consumers’ growing interest in healthy lifestyles. Whole grain consumption offers several nutritional benefits, including lowering total cholesterol and low-density lipoprotein levels [3] and reducing the risk of type 2 diabetes, cardiovascular issues, and intestinal disorders [4]. Wheat kernels comprise three anatomical components: the bran, germ, and endosperm. WWFs, produced by milling the entire kernel without separation of the bran and germ, retain a substantially higher concentration of nutritional constituents—including dietary fiber, essential minerals, vitamins, phytochemicals, and other bioactive compounds—relative to refined flour, which primarily contains the endosperm [5]. However, the nutritional functionality and processing capability of WWFs can vary according to several factors such as wheat variety, particle size, and milling method [6]. In particular, the bran particle size of WWFs notably affects product quality [7]. Numerous studies have examined the impact of WWF particle size on the quality characteristics of flour and noodles, including water absorption, cooking yield, solids leached into the cooking water, polyphenol content, and digestibility [8,9,10].

Milling significantly influences both the particle size distribution and starch characteristics of WWFs, which in turn affect their functional properties such as water absorption and paste viscosity, and suitability for various product applications [5]. As the particle size of wheat flour decreases, the degree of starch damage typically increases due to the greater mechanical forces applied during milling, which disrupt starch granules [11,12]. Siliveru et al. [13] reported that reducing flour particle sizes from 75 to 106 μm to less than 45 μm significantly increased starch damage and decreased powder flowability due to the aggregation of flour particles.

Stone and roller mills are commonly used for producing commercial WWFs [14,15]. However, various laboratory-scale mills such as hammer, ball, cutting, ultra-centrifugal, and cyclone mills have been employed to investigate the impact of particle size on the quality and processing capabilities of WWFs. Compared with commercial mills, these laboratory-scale mills require a relatively small amount of wheat kernels and a short time to produce WWFs for further analyses [16]. Among laboratory-scale mills, the mortar mill, consisting of a mortar and a pestle, was used to achieve the first mechanochemical reactions in multicomponent synthesis by grinding the reactants together. The milling action of the mortar mill is driven by the pressure and friction between the materials and the rotating pestle, a process called “grindstone chemistry”. Although the mill is simple and easy to use, its reproducibility is poor because it depends on the physical strength of the operator [17]. Recently, automated laboratory-scale mortar mills have been introduced, which allow for variations in the pestle rotation speed and milling time, resulting in improved reproducibility compared with older models. Additionally, mortar mills generate substantially low heat during milling due to their gentle grinding action, which minimizes thermal degradation of heat-sensitive constituents and helps preserve the nutritional and functional integrity of the flour (information provided by the mill manufacturer). Amer et al. [18] reported that wheat porridge prepared from flour milled using a traditional mortar-and-pestle technique exhibited lower sugar bioaccessibility compared to porridge prepared from flour milled using modern roller or cyclone mills. Unlike hammer or roller mills, particle size can be controlled without the need for interchangeable sieves, and the simple design of mortar mills allows for easier cleaning and maintenance. Although mortar mills are not widely adopted in large-scale industrial wheat flour production, they can serve as a viable alternative for WWF production, particularly in specialized applications where high-quality, nutritionally rich flours are required in small batches.

However, information regarding the quality characteristics of mortar-milled WWFs is limited. Therefore, in this study, we aimed to investigate the impact of the milling conditions of a mortar mill on the quality characteristics of WWFs, such as particle size distribution, solvent-retention capacity (SRC), starch- and protein-related properties, and nutritional value to evaluate their potential application in WWF and noodle production.

## 2. Materials and Methods

### 2.1. Materials

The Hojoong wheat variety used in this study was supplied by the National Institute of Crop Science (Korea). All reagents were of first-grade quality.

### 2.2. Preparation of Mortar-Milled WWFs and Analysis of Their Particle Sizes

The WWFs were milled from wheat kernels with a laboratory-scale mortar mill (MG200, POWTEQ^®^, Beijing, China). The mill’s technical specifications were as follows: pestle diameter (4.5 cm), rotational speed (50–130 rpm), and power supply (220 V, 50/60 Hz). Whole-wheat kernels (100 g) were poured into the mill over one minute and milled for various durations (10, 20, 40, and 60 min), selected based on a preliminary test that showed differences in particle sizes. Milling was conducted at two rotational speeds (50 and 130 rpm), representing the lowest and highest speeds of the mill. The WWFs milled at 50 and 130 rpm were labeled as L and H, respectively. The WWFs milled for 10, 20, 40, and 60 min at each speed were numbered 10, 20, 40, and 60, respectively (designated as L10, L20, L40, and L60, respectively, for Group L and H10, H20, H40, and H60, respectively, for Group H).

The sizes and size distributions of the WWF particles were measured using a particle size analyzer (LS 13 320, Beckman Coulter, Brea, CA, USA), according to the dry method.

### 2.3. Measuring Moisture, Ash, Total Starch, and Damaged Starch Contents of Mortar-Milled WWFs

The moisture and ash contents of the mortar-milled WWFs were determined using the American Association of Cereal Chemists (AACC) methods 44-15.02 and 08-01.01, respectively [19].

The total starch and damaged starch contents of the mortar-milled WWFs were determined using the Total Starch and Starch Damage Assay Kits (Megazyme International, Wicklow, Ireland), respectively.

### 2.4. Analysis of SRC of Mortar-Milled WWFs

The SRC of the mortar-milled WWFs was determined according to AACC Method 56-11.02 [19]. The weight of the solvent retained by the flour after centrifugation was measured. The SRC values measured using four solvents, water, 5% (*w*/*w*) lactic acid, 5% (*w*/*w*) sodium carbonate, and 50% (*w*/*w*) sucrose, serve as an index to predict flour functionality related to processing and product quality [20]. In this study, the SRC values were measured using two solvents: water, which indicates the overall water-holding capacity of flour, and sodium carbonate, which indicates the levels of damaged starch. Five grams of the flour sample were placed in a 50 mL tube. Subsequently, 25 mL of distilled water or 5% sodium carbonate (Duksan Pure Chem. Co. Ltd., Ansan, Republic of Korea) solution.

### 2.5. Analysis of SDS Sedimentation Volume of Mortar-Milled WWFs

The SDS sedimentation volume of the mortar-milled WWFs was measured according to AACC Method 56-70.01 [19]. First, 5 g of each sample were added to a 100 mL graduated cylinder containing 50 mL of distilled water, with the cylinder capped. The cylinders were vigorously shaken horizontally for the first 15 s and subsequently inverted 10 times within 15 s at 2, 4, and 6 min to ensure complete hydration. After the fourth shaking, 50 mL of SDS–lactic acid reagent (3% SDS in 1.2 N lactic acid) (Sigma-Aldrich, Milwaukee, WI, USA) was added to each cylinder. The cylinders were shaken again, as in the previous step, and inverted 10 times within 15 s at 0, 2, 4, and 6 min. After the final shaking, the cylinders were left upright and the sedimentation volume was recorded at 20, 40, and 60 min.

### 2.6. Analysis of the Mixograph Properties of Mortar-Milled WWFs

The dough properties were measured using a 10 g mixograph (National Manufacturing Co., Lincoln, NE, USA) according to AACC Method 54-40.02 [19]. Flour samples (10 g) and distilled water (6.0 and 6.3 g) were added to the mixing bowl of the mixograph, which was run for 10 min. The mixogram results were recorded.

### 2.7. Analysis of Total Phenolic Content (TPC) and Antioxidant Activity in Mortar-Milled WWFs

The TPC and antioxidant activity of the mortar-milled WWFs were analyzed according to the method described by Yu and Beta [21] with slight modifications. The phenolic extract was obtained from 2 g of flour extracted twice with 10 mL of 80% methanol (99.8%, Duksan Pure Chem. Co. Ltd., Ansan, Republic of Korea) for 60 min using a rotator (CN/VM-80, Miulab, Hangzhou, China), followed by sonication at 0 °C and 40 kHz for 15 min. The flour mixture was then centrifuged for 15 min at 12,000× *g* and 4 °C, and the supernatant was filtered using 90 mm filter paper (No. 2, ADVATEC, Tokyo, Japan). The filtered supernatant was adjusted to a volume of 25 mL with 80% methanol and stored at −20 °C for the analysis of TPC and ABTS radical-scavenging activity.

For measuring TPC, 0.2 mL of flour extract and 1.5 mL of Folin–Ciocalteu reagent (1.9-2.1 N, Sigma-Aldrich, Milwaukee, WI, USA) were reacted for 5 min and neutralized with 1.5 mL of sodium carbonate solution (60 g/L) at 20–25 °C for 90 min. The absorbance of the reaction mixture was measured at 725 nm using a spectrophotometer (X-ma 6100 PC, Human Corporation, Seoul, Republic of Korea). A standard curve was generated using different concentrations of gallic acid (Sigma, St. Louis, MO, USA), and the TPC of the flour was expressed as mg gallic acid equivalents (GAE) per 100 g.

For measuring the ABTS radical-scavenging ability, 10 mL of ABTS reagent was diluted with approximately 990 mL of distilled water. The phenolic extract (50 μL) and diluted ABTS reagent (1.85 mL) were mixed. The absorbance of the reaction mixture at t = 30 min was measured at 734 nm using a spectrophotometer after 30 min in an 80% methanol blank. For absorbance at t = 0 min, 1.85 mL of the diluted ABTS reagent was added to 100 μL of 80% methanol. A standard curve was generated by plotting different Trolox concentrations versus %ABTS decolorization, and the ABTS value was expressed as μL Trolox equivalents (TE)/100 g sample.

### 2.8. Preparation of Noodles Using Mortar-Milled WWFs and Evaluation of Noodle Quality

Noodles were prepared as described by Wang and Kweon [22], with slight modifications. The WWF sample (100 g) was placed in a micropin mixer bowl. Distilled water (36.7–61.9 g) was added, adjusted according to noodle dough consistency (firm, slightly tacky) (water adjustment I: 36.7–44.5 g, relatively low water level) and the water SRC value of the flour (water adjustment II: 38.0–61.9 g, relatively high water level). These water adjustments were applied to assess the effect of adequately wetting the bran on noodle texture. In addition, salt (2 g) was added to each sample. The mixture was mixed for 15 min using a micropin mixer (100 g; National Manufacturing Co., Lincoln, NE, USA). The mixed dough was placed in a plastic bag and allowed to rest for 30 min in a chamber (Phantom M301 Combi, Samjung, Gyeonggi, Republic of Korea) at 35 °C and 85% relative humidity. Subsequently, the dough was continuously sheeted to thicknesses of 3.0, 2.0, and 1.5 mm and then cut into noodle strands (15 × 35 mm) using a noodle maker (SN-88, Samwoo Industrial Co., Daegu, Republic of Korea). The color of fresh noodles was measured using a colorimeter (CR-20, Konica Minolta, Tokyo, Japan) to analyze the dough sheet before it was cut into noodles. The resistance (R) and extensibility (E) of fresh noodles (2.0 mm width × 1.5 mm thickness) were measured using a texture analyzer (CT3, Brookfield, Middleboro, MA, USA). The measurement conditions were as follows: probe, Kieffer rig (TA-KF); test mode, tension; target value, 20 mm; pre-test speed, 2 mm/s; and test speed, 3.3 mm/s. The resistance to extensibility ratio (R/E) was calculated using the measured resistance and extensibility values.

To measure the quality of the cooked noodles, fresh noodles (15 g) were cooked in boiling water (500 mL) for 15 min. The cooking water was drained into a beaker, and the turbidity was measured using a spectrophotometer at 675 nm. Weight gain (%) during cooking was calculated as the difference between the weight of fresh noodles before cooking and that of cooked noodles before rinsing. For analyzing textural characteristics, the cooked noodles were lightly rinsed with tap water to cool them to room temperature, gently shaken on a sieve to remove the water, and analyzed immediately, as described by Wang and Kweon [22]. Five strands of cooked noodles (6 cm in length) were aligned on the plate of an Asian noodle rig (TA 7) and analyzed using a texture analyzer under the following conditions: test mode, texture profile analysis; deformation, 70%; pre-test speed, 2 mm/s; test speed, 1 mm/s; and post-test speed, 1 mm/s. Firmness, adhesiveness, springiness, cohesiveness, and chewiness were analyzed.

### 2.9. Statistical Analysis

All data were obtained from at least duplicate experiments, and variations in flour and noodle quality were analyzed using Tukey’s HSD test with SPSS Statistics (ver. 27.0; IBM, Armonk, NY, USA). Differences were considered statistically significant at *p* < 0.05.

## 3. Results and Discussion

### 3.1. Appearance and Particle Size Distribution of WWFs

**Figure 1:**
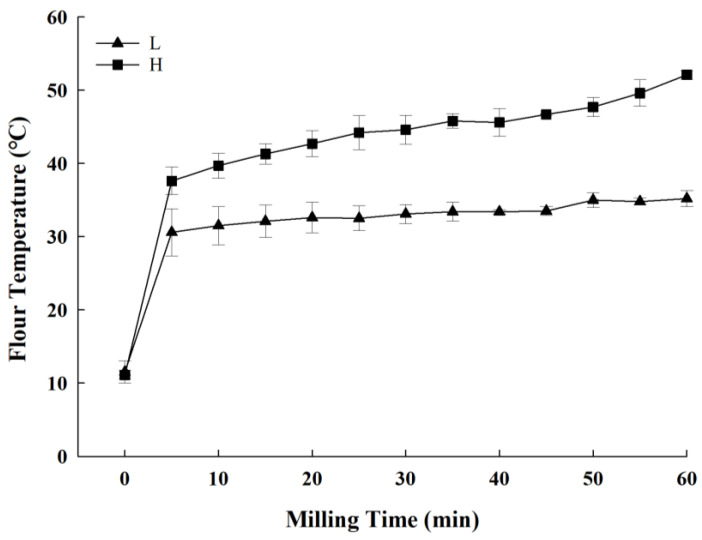
Temperature progression over time in whole-wheat flours (WWFs) milled in a mortar mill: “L” represents WWFs milled at 50 rpm (Group L), and “H” represents WWFs milled at 130 rpm (Group H).

The flour temperatures measured during milling are shown in Figure 1. The WWFs milled at high pestle speeds (Group H) exhibited a significant increase in temperature, whereas those milled at low pestle speeds (Group L) showed no significant increase. After 5 min of milling, Group L showed an approximate 30 °C increase in flour temperature, which remained stable or slightly increased over 60 min. In contrast, the temperature for Group H increased to 38 °C at 5 min and continued to increase to 50 °C by 60 min. Prabhasankar and Haridas Rao [23] reported the temperature of WWF milled using four different types of mills—stone, plate, hammer, and roller. They observed increases in flour temperature (35–55 °C) with the hammer and roller mills; however, the protein structure remained intact in SDS-PAGE patterns due to minimal heat penetration. In contrast, much higher temperature increases (85–95 °C) were observed with the stone and plate mills, leading to protein degradation and significant loss of total amino acids. Thus, the temperature rises observed during mortar milling closely matched those reported for hammer and roller mills. Due to the small scale of the laboratory mill, protein denaturation was unlikely to occur—an outcome that benefits the mortar milling process.

**Figure 2:**
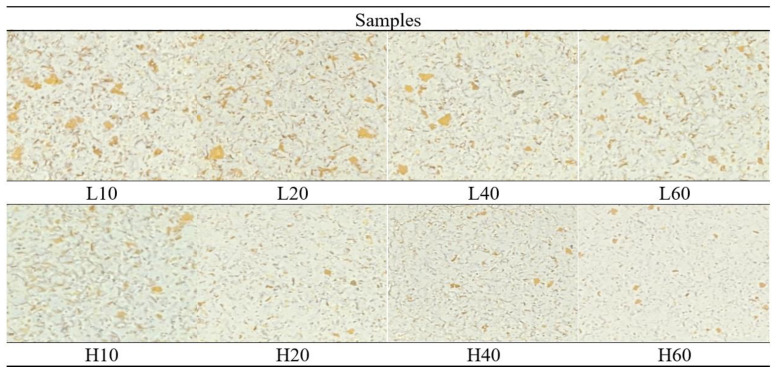
Appearance of the mortar-milled WWFs: L10, L20, L40, and L60 represent WWFs milled at 50 rpm for 10, 20, 40, and 60 min, respectively; H10, H20, H40, and H60 represent WWFs milled at 130 rpm for 10, 20, 40, and 60 min, respectively.

The appearance of the mortar-milled WWFs is shown in Figure 2. Samples L10 through L60 exhibited considerably larger speckles from the bran than exhibited by samples H10 through H60, indicating a significant variation in the particle size of WWFs produced under different milling conditions. The L60 WWF, milled at a low rotating pestle speed for a long duration, appeared similar to the H10 WWF, milled at a high rotating pestle speed for a short duration. This result suggests that an equivalent amount of work or energy input was applied to the wheat kernels during the milling of both WWFs, leading to similar grindability and particle sizes.

**Figure 3:**
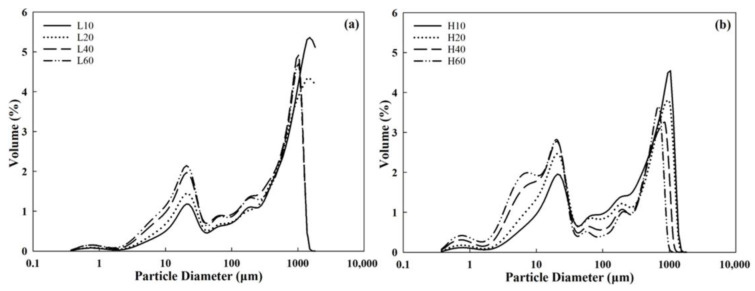
Particle size distribution of the mortar-milled WWFs: (**a**) Group L (**b**) Group H. L10, L20, L40, and L60 represent WWFs milled at 50 rpm for 10, 20, 40, and 60 min, respectively; H10, H20, H40, and H60 represent WWFs milled at 130 rpm for 10, 20, 40, and 60 min, respectively.

The particle size distributions of the mortar-milled WWFs are presented in Figure 3, and the average particle sizes (d10, d50, and d90) are listed in Table 1. Compared with Group H, Group L exhibited a substantially different particle size distribution pattern, with a considerably larger proportion of large particles, as indicated by the smaller peak area of the first peak. As the milling time increased, the area of the first peak expanded, indicating particle size reduction. The d50 values for L10, 20, 40, and 60 were 692, 552, 246, and 203 μm, respectively, whereas the values for H10, 20, 40, and 60 were 250, 143, 34, and 24 μm, respectively. These results indicate that a higher pestle speed had a more pronounced impact on particle size reduction during milling with increasing milling time, as greater mechanical forces were applied to the wheat kernels. Factor analysis of WWFs (pestle speed at two levels; milling time at four levels) on particle size, performed in Design-Expert v13 (Stat-Ease, Minneapolis, MN, USA), revealed that pestle speed significantly influences d10, d50, and d90 (*p* < 0.05), whereas milling time only significantly affects d10 (*p* < 0.05). In a previous study on the quality of commercial WWFs [24], mortar-milled WWFs exhibited a higher proportion of larger particles (with a significantly larger d90) than most WWFs, except for WWFs produced in France, indicating a considerably broader particle size distribution.

### 3.2. Moisture, Ash, Total Starch, and Damaged Starch Contents of WWFs

The moisture and ash contents of the WWFs are listed in Table 2. The moisture content of the WWFs was higher in Group L than in Group H for the same milling times. As the milling time increased, the moisture content of the WWFs decreased owing to increased drying. Group H showed a more pronounced reduction in the moisture content of the WWFs than showed by Group L. During the milling process, the mortar mill generates pressure and frictional heat through the rotating pestle, causing the moisture in the flour samples to evaporate. van Rooyen et al. [25] reported that hammer and stone milling, which involve physical damage to the endosperm through friction, cause the grains to heat and lose moisture. Consequently, the moisture content was the lowest for the H60 WWFs, milled under the most intensive conditions (high pestle speed and long milling time). Conversely, the ash content showed no considerable difference or only a slight increase with long milling times, which is consistent with the outcomes for the same wheat variety used for WWF preparation as shown in a previous study [26].

The total and damaged starch contents of the WWFs are presented in Table 2. The damaged starch generated during milling considerably increases the water-holding capacity of the flour, which negatively affects the quality of cookies and crackers [27]. An increase in milling time led to a corresponding increase in both total starch and damaged starch contents, confirming the influence of milling force and work on the physical alteration of starch granules. H60 WWF, subjected to the highest milling energy, exhibited the highest total starch and damaged starch contents (59.2% and 10.6%, respectively). This result is consistent with the findings of Ranhotra et al. [28], who observed an increase in damaged starch levels with prolonged milling. Additionally, the total starch content increased as the particle size decreased, as observed in oat flour, where the total starch content increased from 34.3% to 69.9% with decreasing sieve aperture [29]. Similarly, L60 and H10, which had comparable particle sizes, showed equivalent levels of total (55.4%) and damaged (3.3–3.5%) starch, likely reflecting comparable mechanical-energy inputs. Under equivalent milling durations, WWFs in Group H showed significantly higher damaged-starch contents (3.3–10.6%) than those in Group L (2.0–3.5%) (*p* < 0.05), indicating greater mechanical-energy input in Group H. Niu et al. [30] reported increased starch damage caused by superfine grinding, which reduced the particle size of WWFs. The correlations between damaged starch and particle sizes (d10, d50, and d90) in this study were significant, with values of r = −0.83**, −0.75*, and −0.80*, respectively (*, *p* < 0.05; **, *p* < 0.01).

### 3.3. SRC of WWFs

**Figure 4:**
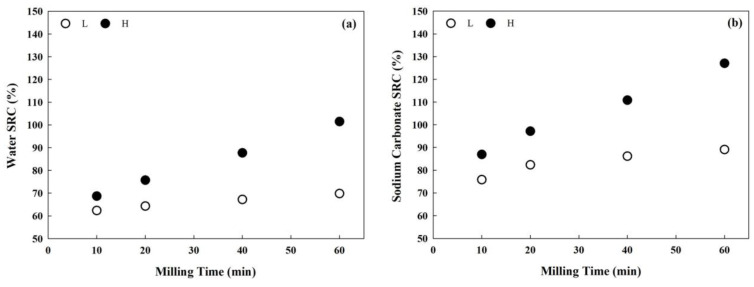
Solvent-retention capacity (SRC) curves of the mortar-milled WWFs in water (**a**) and sodium carbonate solution (**b**): “L” represents WWFs milled at 50 rpm (Group L), and “H” represents WWFs milled at 130 rpm (Group H).

The SRC results for the mortar-milled WWFs are shown in Figure 4. The impact of increased milling time on water and sodium carbonate SRCs was greater in Group H, with smaller particle sizes, than in Group L, which had larger particle sizes. The water SRC values for Groups L and H ranged from 62.3% to 69.8% and from 68.7% to 101.5%, respectively. The sodium carbonate SRC values for Groups L and H ranged from 75.8% to 89.1% and from 86.9% to 127.0.5%, respectively. Sodium carbonate SRC is related to the damaged starch content [19]. These results suggest that more damaged starch was generated during milling at higher pestle speeds for long durations. Damaged starch generally absorbs more water than undamaged starch, leading to increased water SRC. Additionally, the sodium carbonate SRC, which is influenced by the swelling of damaged starch granules according to damage severity, increased with higher pestle speed and longer milling durations. Barak et al. [31] reported that the water and sodium carbonate SRC values of two wheat varieties considerably increased as their flour particle sizes decreased, consistent with the results of the present study. The sodium carbonate SRC results corresponded to the trend observed for the damaged starch content shown in Table 2. Water and sodium carbonate SRCs were considerably correlated with the particle size (d10, d50, and d90). The correlation coefficients between water SRC and particle sizes were r = −0.85**, −0.77*, and −0.81**, respectively (*, *p* < 0.05; **, *p* < 0.01). Similarly, the correlation coefficients between sodium carbonate SRC and particle sizes (d10, d50, and d90) were r = −0.89**, −0.81**, and −0.84** (**, *p* < 0.01), respectively.

### 3.4. Pasting Properties of WWFs

**Figure 5:**
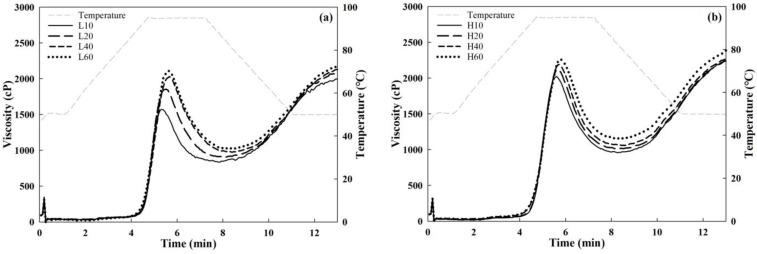
Pasting curves of the mortar-milled WWFs, as determined using a rapid viscosity analyzer: (**a**) Group L (**b**) Group H. L10, L20, L40, and L60 represent WWFs milled at 50 rpm for 10, 20, 40, and 60 min, respectively; H10, H20, H40, and H60 represent WWFs milled at 130 rpm for 10, 20, 40, and 60 min, respectively.

The pasting patterns determined using the rapid viscosity analyzer (RVA) are shown in Figure 5, and the pasting property parameters are presented in Table 3. Most RVA viscosity parameters were higher in Group H than in Group L. As the milling time increased, the pasting viscosities, including the peak, breakdown, final, and setback viscosities, increased.

The pasting properties of refined wheat flour are influenced by damaged starch content [32]; as the damaged starch content increases, the viscosity decreases [33]. In refined wheat flour, particle size reduction lowers the pasting temperature, allowing easier penetration of water molecules into starch granules and cleavage of intermolecular hydrogen bonds in wheat starch during heating, which results in the destruction of crystalline regions within the granules [11]. As particle size decreases, gelatinization typically occurs at lower temperatures, leading to a decrease in pasting temperature [34]. However, this study presented a conflicting trend because the flour tested in previous studies was refined wheat flour, whereas this study used WWF; in WWF, bran particle size, in addition to starch damage, plays a remarkable role in gelatinization. WWFs contain a considerable portion of bran, and in particular, large bran particles can negatively affect the increase in viscosity during pasting. Islam et al. [35] reported that reducing the particle size of stone-milled WWF from 151 to 117 μm increased starch damage from 6.58% to 6.71%, leading to higher peak, breakdown, and final viscosities. These higher viscosities were attributed to facilitation of granular swelling and starch gelatinization, which increased the consistency of starch paste [36]. The pasting temperature decreased as the milling time increased at both low and high pestle speeds. In this study, significant correlations were observed between peak viscosity and particle sizes (d10, d50, and d90), with r = −0.97***, −0.98***, and −0.94***, respectively (***, *p* < 0.001). Similarly, the correlations between final viscosity and particle sizes (d10, d50, and d90) were significant, with r = −0.96***, −0.94**, and −0.94***, respectively (**, *p* < 0.01; ***, *p* < 0.001).

### 3.5. SDS Sedimentation Volume of WWFs

The SDS sedimentation volumes of the WWFs are listed in Table 4. The SDS sedimentation volume of Group L did not show any significant change over time, whereas that of Group H increased. Additionally, as the particle size of the WWFs decreased, Group L showed no significant change in the SDS sedimentation volume from L10 to L60, whereas Group H showed a decrease from H10 to H60.

Previous studies have reported a correlation between SDS sedimentation volume and flour protein quality [37,38]. However, reduced particle size in refined flour can lower the SDS sedimentation volume [39], highlighting the impact of particle size. In our study, the relationship between the SDS sedimentation volume at 20 min and particle sizes (d10, d50, and d90) was significant, with r = 0.81, 0.74, and 0.75, respectively (*p* < 0.05). The lower SDS sedimentation volume of Group H compared to that of Group L may be attributed to the significantly slower settling of the WWFs because of decreased particle size rather than flour protein quality. The low settling speed may explain the increase in SDS sedimentation volume over time in Group H. These results underscore the importance of considering particle size when interpreting the SDS sedimentation volume of WWFs in gluten quality analysis.

Navarro et al. [16] investigated the effect of WWF particle shape on SDS sedimentation volume using various mills, including cyclonic, hammer, and roller mills. They found that flour particles with an enhanced amount of endosperm attached to the bran impeded protein unfolding by SDS and formed small but heavier floccules, which resulted in a lower SDS sedimentation volume. Similarly, Morris et al. [40] reported that the grinder type and screen aperture (particle size) considerably affected the SDS sedimentation volume of wheat meal from 10 wheat varieties; however, this effect was less pronounced than that of wheat variety.

Furthermore, increased flour temperatures of approximately 85–90 °C, generated during milling, especially in stone and plate mills, caused protein denaturation, particularly of high-molecular-weight glutenin proteins [23]. However, the flour temperature in this study was not high (Figure 1), suggesting that glutenin protein denaturation was unlikely to have played a role.

### 3.6. Dough Mixing Properties of WWFs

**Figure 6:**
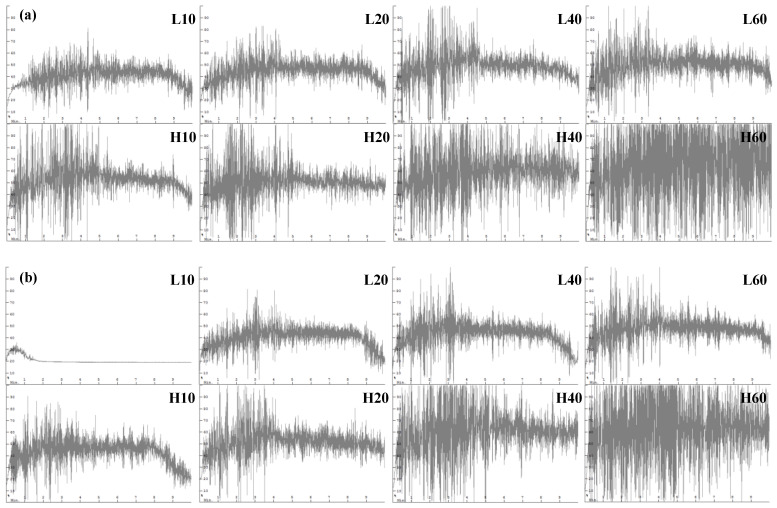
Mixograms of the mortar-milled WWFs: (**a**) 6.0 g of water added; (**b**) 6.3 g of water added; L10, L20, L40, and L60 represent WWFs milled at 50 rpm for 10, 20, 40, and 60 min, respectively; H10, H20, H40, and H60 represent WWFs milled at 130 rpm for 10, 20, 40, and 60 min, respectively.

The mixograms of the WWFs are shown in Figure 6. Compared to Group L, Group H exhibited larger mixing bandwidths at both water levels, with a more pronounced effect at the higher water level (6.3 g). These results indicate that Group H exhibited greater gluten strength than Group L, with pestle speed having a more pronounced effect than milling duration under the milling conditions tested.

With increasing milling time, both groups showed an increase in the mixing bandwidth and less significant dough weakening during mixing, as indicated by the reduced slope of the mixing band. In particular, L10 in Group L, which was characterized by a large particle size, low damaged starch content, and low water-retention capacity, was too wet to form dough at the higher water level. In contrast, H40 and H60, which had smaller particle sizes and higher damaged starch contents, required more water and a longer mixing time for dough development. Although these samples exhibited no marked dough weakening, the water was insufficient for hydrating the flour components. Factor analysis of WWFs under different milling conditions showed that both pestle speed and milling time significantly influenced the midline peak height of dough mixed with 6.0 and 6.3 g of water (*p* < 0.05). The correlation coefficients between particle sizes (d10, d50, and d90) and midline peak height varied depending on milling conditions. For doughs mixed with 6.0 g of water, the correlations were r = −0.94***, −0.94***, and −0.96***, respectively; for doughs mixed with 6.3 g of water, they were r = −0.98***, −0.96***, and −0.92***, respectively (***, *p* < 0.001). Additionally, the midline peak time and peak width of dough mixed with 6.3 g of water were also significantly influenced by particle size.

Correlation analysis indicated that extremely low pestle speed and short milling time, or excessively high pestle speed and extended milling time, may be undesirable. These extremes can result in overly large particles, leading to low peak height, or excessive starch damage, resulting in high peak height and water absorption during dough mixing.

Consequently, the L40 and L60 samples—produced using a low pestle speed with longer milling durations—and the H10 and H20 samples—prepared at a high pestle speed with shorter durations—yielded doughs with similar mixing patterns despite variations in water levels, suggesting that these combinations may be more desirable.

Bressiani et al. [41] reported an increase in dough stability of WWF as the particle size decreased from 830.0 to 194.9 μm. This suggests that finer whole-grain particles enhance resilience during mixing and contribute to stronger dough development. Similarly, Lin et al. [42] observed that particle size reduction of WWF resulted in considerable increases in stability time, dough development time, and time to breakdown, along with a decrease in the mixing tolerance index in the farinograph. This indicates that, compared with coarse particles (mean size: 1350 μm), fine particles (mean size: 199 μm) exhibited an increased dough strength and reduced destructive effect on the gluten network, resulting in dough with the highest resistance to mechanical damage.

However, other studies have reported that a decrease in WWF particle size results in either no notable changes [29,43] or a decrease in dough development time, stability, and gluten strength [44,45]. These discrepancies are likely due to differences in particle size ranges and wheat varieties, as the latter studies primarily examined smaller maximum particle sizes or narrower particle size distributions.

### 3.7. TPC and Antioxidant Activity in Mortar-Milled WWFs

TPC and the antioxidant activities of the mortar-milled WWFs, based on ABTS radical-scavenging activity assays, are shown in Table 5. As particle size decreased within each group (L and H), TPC increased as follows: L10 < L20 < L60 ≤ L40 in Group L and H10 < H20 ≤ H40 ≤ H60 in Group H. Milling conditions significantly influenced TPC, which was higher in WWFs prepared under higher pestle speeds that resulted in smaller particle sizes. This trend aligns with previous findings that finer wheat aleurone, bran, and WWF have higher TPC values [46,47,48], possibly because of the increased surface area, which enhances phenolic compound extraction. Rosa et al. [49] reported that reducing the particle size increased the antioxidant activity, as high-energy grinding tripled the specific surface area of wheat bran, leading to greater exposure to aleurone cell wall phenolic acids.

However, ABTS radical-scavenging activity followed an opposite trend in Group L (L60 ≤ L40 < L20 ≈ L10) and showed no significant difference in Group H (H40 ≤ H60 ≤ H20 < H10). The milling conditions affected the ABTS radical-scavenging activity in Group L, wherein the longer milling time resulted in lower activity. Contrastingly, the WWFs milled at higher pestle speeds exhibited no significant differences in ABTS radical-scavenging activity for different milling times. Antioxidant activity is influenced not only by polyphenols but also by other compounds, such as flavonoids, carotenoids, and ferulic acid [46], which may explain why the TPC and ABTS results do not always correlate. Similarly, Brewer et al. [50] observed an increase in TPC but a decrease in DPPH scavenging activity after ultrafine grinding of wheat bran; this result was attributed to differences in the redox potential between molybdenum in the Folin–Ciocalteu reagent and DPPH. Phenolic compounds are heat-labile and prone to degradation during storage and processing, which can alter their biological activity [51]. The temperature rise observed during mortar milling in this study may also affect these compounds and their antioxidant properties, thereby contributing to variability in total phenolic content (TPC) and ABTS assay results. Although pestle speed and milling duration in this study did not significantly affect TPC and ABTS (*p* > 0.05), significant correlations were observed between TPC and particle sizes (d10, d50, and d90), with correlation coefficients of r = −0.84**, −0.85*, and −0.88*, respectively (*p* < 0.01). Similarly, ABTS values were significantly correlated with particle sizes, showing coefficients of r = 0.96***, 0.97***, and 0.95*** for d10, d50, and d90, respectively (*p* < 0.001).

### 3.8. Color and Textural Properties of Fresh Noodles Made from Mortar-Milled WWFs

The color of fresh noodles prepared from mortar-milled WWFs is presented in Table 6. Lightness (L*), redness (a*), and yellowness (b*) values showed no significant trends in either Group L or Group H, indicating that particle size, as influenced by different mortar milling conditions, did not significantly affect noodle color. These results suggest that although the water absorption capacity of the WWFs remained similar in Group L, it increased in Group H with longer milling times. In contrast, increased water content during fresh noodle preparation significantly impacted noodle color, decreasing L* values but increasing a* and b* values. Hatcher et al. [52] reported that a higher water content decreased L* values and increased b* values in noodles, consistent with our findings.

The resistance of fresh noodles prepared using water adjustment I (36.7–44.5 g) increased from 0.28 to 0.37 N in Group L but decreased from 0.39 to 0.33 N in Group H. Contrastingly, extensibility showed no significant trend in Group L (from 2.32 to 2.25 mm) but decreased significantly in Group H (from 2.85 to 1.75 mm). For noodles prepared using water adjustment II (38–61.9 g), resistance significantly increased from 0.33 to 0.41 N in Group L but significantly decreased from 0.39 to 0.13 N in Group H. Extensibility followed a similar trend, increasing in Group L (from 2.52 to 2.70 mm) but significantly decreasing in Group H (from 2.89 to 1.01 mm). These results suggest that noodles prepared with H40 and H60 were excessively wet due to excessive water addition. The greater variations in the textural properties of these noodle doughs between water adjustments I and II are likely caused by the amplified effect of damaged starch generated during bran particle reduction on water-holding capacity. Using water SRC to adjust water levels for noodle preparation (such as in water adjustment II) is effective and easily applicable to noodles made from refined wheat flour. However, for noodles prepared with WWF, careful consideration is required due to variations in bran particle size, which make water level adjustment more challenging.

Bran in WWFs interrupts gluten network formation and reduces gluten matrix continuity by diluting gluten, competitive water absorption, and fiber–gluten interaction [53,54]. Factor analysis of milling conditions revealed that pestle speed significantly affected the R/E ratio of fresh noodles prepared using water adjustment I. Although pestle speed and milling duration did not significantly affect color and resistance and extensibility of fresh noodles (*p* > 0.05), significant correlations were observed between noodle color and particle sizes (d10, d50, and d90), with correlation coefficients of r = 0.88**, 0.86**, and 0.85**, respectively (*p* < 0.01). Likewise, R/E ratios were significantly correlated with particle sizes, showing coefficients of r = −0.83*, 0.76*, and 0.78* for d10, d50, and d90, respectively (*p* < 0.05). Consistent with observations from dough mixing properties, the L60 and H10 noodle samples exhibited smaller variations in color and textural parameters across different water adjustments. This suggests that WWFs produced under either low pestle speed with extended milling duration or high pestle speed with shorter duration may be more suitable for noodle production.

During noodle production, the sheeting and cutting processes require well-balanced dough with good extensibility and resistance to form smooth noodle sheets and prevent breakage. This balance depends on adequate gluten strength [55,56]. Therefore, controlling the WWF particle size and water addition can effectively modify dough resistance and extensibility.

### 3.9. Quality Characteristics of Cooked Noodles

**Figure 7:**
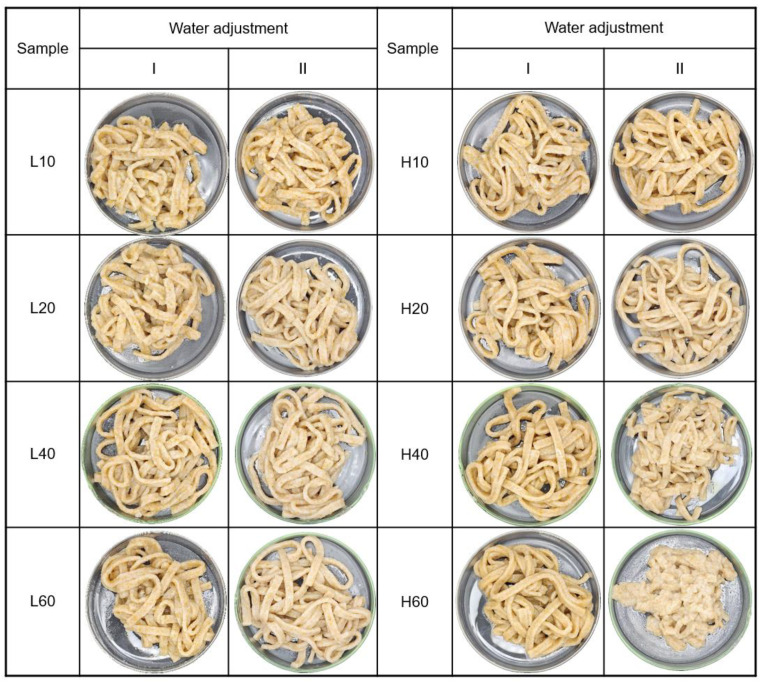
Appearance of cooked noodles prepared using mortar-milled WWFs. L10, L20, L40, and L60 represent WWFs milled at 50 rpm for 10, 20, 40, and 60 min, respectively; H10, H20, H40, and H60 represent WWFs milled at 130 rpm for 10, 20, 40, and 60 min, respectively.

The appearance of the cooked noodles is shown in Figure 7. Fresh noodles prepared from L10 and L20 WWFs exhibited noticeable bran speckles, whereas those prepared from H40 and H60 did not show large bran speckles. In comparison, cooked noodles displayed fewer bran speckles because of hydration and wetting of the bran during cooking.

For noodles prepared using water adjustment I (36.7–44.5 g), cooked noodles from L10 and L20 appeared mushy and soggy, whereas those made from H40 and H60 were relatively springy and firm, indicating that gluten development during noodle making influences their texture. In contrast, for noodles prepared using water adjustment II (38.0–61.9 g), cooked noodles from H40 and H60 appeared lumpy and soggy, whereas those made from L10 and L20 remained relatively springy and firm, highlighting the impact of water content on cooked noodle quality. As described previously, adjusting the water level for noodles prepared using WWF requires careful consideration because of the complex dough-development process, which is influenced by bran particle size and distribution.

The weight gain and turbidity of the cooking water for noodles made from mortar-milled WWFs are presented in Table 7. Weight gain showed no significant difference with particle-size reduction, except for the H40 and H60 noodles prepared with water adjustment II. These noodles exhibited a significantly lower weight gain, likely because excessive wetness caused the solid materials to leach during cooking, thereby increasing the turbidity of the cooking water. In contrast, turbidity decreased slightly with decreasing WWF particle size.

The textural characteristics of the cooked noodles are presented in Table 7. The texture of noodles prepared with H60 WWF and water adjustment II was not analyzed because they were too lumpy to separate after cooking. For cooked noodles prepared using water adjustment I, firmness increased in the order of L10 ≈ L20 < L60 ≤ L40 in Group L and H10 ≤ H20 < H60 < H40 in Group H. On the contrary, for cooked noodles prepared using water adjustment II, firmness increased in the order of L10 < L20 < L40 ≤ L60 in Group L, whereas the order was H40 < H20 < H10 in Group H.

The resilience, cohesiveness, and springiness of cooked noodles with water adjustment I followed similar trends. Although Group L showed no significant differences in these characteristics, in Group H, these properties considerably increased as the particle size decreased with longer milling times. Similarly, Niu et al. [24] reported notable increases in hardness, cohesiveness, and resilience in cooked WWF noodles as particle size decreased from 125 to 43 μm. For noodles prepared with water adjustment II, these textural properties increased in groups L and H but not in H40.

Chewiness, which reflects the mouthfeel [57], followed a trend similar to that of firmness. In cooked noodles prepared using water adjustment I, chewiness increased in the order of L10 ≈ L20 ≤ L40 ≈ L60 in Group L, whereas the order was H10 ≤ H20 < H40 ≈ H60 in Group H. For cooked noodles prepared using water adjustment II, chewiness increased in the order of L10 ≤ L20 < L40 ≤ L60 in Group L, whereas the order was H40 < H20 ≈ H10 in Group H. The cooked noodles prepared with L40, L60, H10, and H20 WWFs showed relatively small variations between water adjustments I and II while exhibiting high firmness, springiness, and chewiness. Factor analysis of milling conditions revealed that pestle speed significantly affected the firmness, springiness, and chewiness of cooked noodles prepared using water adjustment I (*p* < 0.05). These textural properties showed significant correlations with particle size parameters (d10, d50, and d90). Specifically, firmness was negatively correlated with particle size (r = −0.93**, −0.93**, and −0.93**, respectively; *p* < 0.01); springiness was also negatively correlated (r = −0.91**, −0.84**, and −0.86**, respectively; *p* < 0.01); and chewiness exhibited similar negative correlations (r = −0.93**, −0.88**, and −0.89**, respectively; *p* < 0.01).

Chen et al. [58] reported a decrease in the hardness and chewiness of cooked dry white Chinese noodles as the levels of added bran and particle size increased. Park and Baik [59] found that water absorption affects the firmness of both fresh and cooked noodles. Hou et al. [60] observed that firmness, springiness, and chewiness were negatively correlated with dough extensibility but positively correlated with dough resistance, consistent with the findings of this study.

Overall, the lab-scale mortar mill is time-consuming for WWF preparation but serves as a useful tool for comparing the effects of particle size and damaged starch on noodle quality using small sample amounts. A higher pestle speed and shorter milling time may be more suitable for WWF production, considering energy costs, though future validation is required.

## 4. Conclusions

In this study, we have demonstrated the notable effects of mortar milling conditions, particularly pestle speed and milling duration, on the physicochemical properties of WWF. Pestle speed significantly influenced flour temperature, particle size (d10, d50, and d90), sucrose SRC, pasting viscosities and temperatures, SDS sedimentation volume, and cooked noodle texture. Milling duration affected particle size (d10), final pasting viscosities and temperature, and cooked noodle firmness.

Higher pestle speeds resulted in increased flour temperature; however, the temperature remained within a range unlikely to cause protein denaturation. The particle size distribution varied significantly between low-speed (Group L) and high-speed (Group H) milling, with Group H exhibiting finer particles and a broader distribution.

Increased milling time led to greater moisture loss, higher starch damage, and increased water and sodium carbonate SRC values, particularly in Group H. The pasting properties of the WWFs were strongly affected by particle size, with reduced particle sizes leading to higher pasting viscosities. Additionally, the SDS sedimentation volume was influenced more by particle size than by flour protein quality.

The dough mixing properties and noodle textural characteristics also varied with particle size and water absorption capacity. Group H exhibited stronger gluten networks but required careful water adjustment to prevent excessive hydration. TPC increased with finer particle size; however, the antioxidant activity trends varied depending on the milling conditions.

Overall, this study highlights the utility of a mortar mill for small-batch (lab-scale) WWF production and emphasizes the importance of controlling milling parameters—such as pestle speed and milling duration—to achieve desirable WWF properties, including improved dough performance and noodle quality. These findings offer valuable insights into the application of mortar milling as a feasible alternative for WWF production and provide foundational knowledge for optimizing particle size and ensuring consistent WWF quality prior to scale-up in the milling industry.

## Figures and Tables

**Table 1:** Particle size of the mortar-milled whole-wheat flour (WWF) samples.

Sample	Particle Size (μm)		
	d10	d50	d90
L10	20 ± 0 ^h(1)^	692 ± 10 ^g^	1673 ± 1 ^g^
L20	16 ± 0 ^g^	552 ± 1 ^f^	1605 ± 3 ^f^
L40	11 ± 0 ^e^	246 ± 1 ^d^	1066 ± 2 ^d^
L60	9 ± 0 ^d^	203 ± 3 ^c^	1058 ± 0 ^d^
H10	12 ± 0 ^f^	250 ± 4 ^d^	1051 ± 0 ^d^
H20	8 ± 0 ^c^	143 ± 5 ^b^	1002 ± 10 ^c^
H40	5 ± 0 ^b^	34 ± 1 ^a^	816 ± 8 ^b^
H60	4 ± 0 ^a^	24 ± 0 ^a^	681 ± 1 ^a^

^(1)^ Values (Mean ± SD) with the same letter within the same column are not significantly different (*p* > 0.05) according to Tukey’s honestly significant difference (HSD) test. L10, L20, L40, and L60 represent WWFs milled at 50 rpm for 10, 20, 40, and 60 min, respectively; H10, H20, H40, and H60 represent WWFs milled at 130 rpm for 10, 20, 40, and 60 min, respectively. The parameters d10, d50, and d90 denote the particle diameters corresponding to the 10th, 50th, and 90th percentiles of the cumulative volume distribution, respectively.

**Table 2:** Moisture, ash, total, and damaged starch contents of the mortar-milled WWF samples.

Sample	Moisture Content (%)	Ash Content (%)	Total Starch Content (%)	Damaged Starch Content (%)
L10	13.4 ± 0.1 ^g(1)^	1.48 ± 0.02 ^ab^	51.9 ± 0.0 ^a^	2.0 ± 0.1 ^a^
L20	13.0 ± 0.0 ^f^	1.48 ± 0.02 ^ab^	53.3 ± 0.0 ^b^	2.4 ± 0.2 ^b^
L40	12.2 ± 0.1 ^d^	1.50 ± 0.00 ^abc^	54.7 ± 0.0 ^c^	3.1 ± 0.2 ^c^
L60	12.0 ± 0.0 ^d^	1.55 ± 0.02 ^cd^	55.4 ± 0.2 ^d^	3.5 ± 0.1 ^c^
H10	12.5 ± 0.1 ^e^	1.45 ± 0.02 ^a^	55.4 ± 0.0 ^d^	3.3 ± 0.2 ^c^
H20	11.1 ± 0.1 ^c^	1.53 ± 0.03 ^bcd^	55.6 ± 0.0 ^d^	5.0 ± 0.2 ^d^
H40	7.9 ± 0.1 ^b^	1.57 ± 0.00 ^de^	56.9 ± 0.1 ^e^	7.0 ± 0.2 ^e^
H60	5.7 ± 0.0 ^a^	1.61 ± 0.02 ^e^	59.2 ± 0.0 ^f^	10.6 ± 0.2 ^f^

^(1)^ Values (Mean ± SD) with the same letter within the same column are not significantly different (*p* > 0.05) according to Tukey’s HSD test. L10, L20, L40, and L60 represent WWFs milled at 50 rpm for 10, 20, 40, and 60 min, respectively; H10, H20, H40, and H60 represent WWFs milled at 130 rpm for 10, 20, 40, and 60 min, respectively.

**Table 3:** Pasting properties of the mortar-milled WWF samples, as determined using a rapid viscosity analyzer.

Sample	Peak Viscosity (cP)	Breakdown Viscosity (cP)	Final Viscosity (cP)	Setback Viscosity (cP)	Peak Time (min)	Pasting Temperature (°C)
L10	1578 ± 5 ^a(1)^	747 ± 7 ^a^	1991 ± 8 ^a^	1160 ± 2 ^a^	5.38 ± 0.03 ^a^	90.6 ± 0.8 ^c^
L20	1860 ± 6 ^b^	961 ± 12 ^b^	2074 ± 26 ^b^	1176 ± 13 ^a^	5.49 ± 0.03 ^ab^	90.1 ± 0.3 ^bc^
L40	2043 ± 18 ^c^	1074 ± 13 ^cd^	2130 ± 6 ^bc^	1161 ± 4 ^a^	5.60 ± 0.06 ^bc^	89.3 ± 0.4 ^abc^
L60	2112 ± 13 ^d^	1085 ± 4 ^cde^	2170 ± 11 ^cd^	1143 ± 4 ^a^	5.62 ± 0.03 ^c^	88.5 ± 0.4 ^ab^
H10	2015 ± 20 ^c^	1051 ± 17 ^c^	2220 ± 13 ^de^	1256 ± 15 ^b^	5.62 ± 0.03 ^c^	90.4 ± 0.0 ^c^
H20	2123 ± 14 ^d^	1112 ± 2 ^def^	2249 ± 18 ^ef^	1238 ± 10 ^b^	5.69 ± 0.03 ^cd^	89.6 ± 0.0 ^bc^
H40	2192 ± 1 ^e^	1124 ± 11 ^f^	2308 ± 37 ^f^	1240 ± 26 ^b^	5.71 ± 0.03 ^cd^	88.5 ± 0.5 ^ab^
H60	2251 ± 10 ^f^	1101 ± 8 ^ef^	2377 ± 8 ^g^	1227 ± 5 ^b^	5.80 ± 0.00 ^d^	88.0 ± 0.7 ^a^

^(1)^ Values (Mean ± SD) with different lowercase letters within the same column differed significantly (*p* < 0.05), according to Tukey’s HSD test. L10, L20, L40, and L60 represent WWFs milled at 50 rpm for 10, 20, 40, and 60 min, respectively; H10, H20, H40, and H60 represent WWFs milled at 130 rpm for 10, 20, 40, and 60 min, respectively.

**Table 4:** Sodium dodecyl sulfate (SDS) sedimentation volume of the mortar-milled WWF samples.

Sample	SDS Sedimentation (mL)		
	20 min	40 min	60 min
L10	16.5 ± 0.5 ^c(1)^	16.5 ± 0.5 ^d^	16.5 ± 0.5 ^a^
L20	15.3 ± 0.3 ^c^	15.5 ± 0.5 ^d^	15.5 ± 0.5 ^a^
L40	15.8 ± 0.3 ^c^	16.0 ± 0.0 ^d^	16.0 ± 0.0 ^a^
L60	16.3 ± 0.3 ^c^	16.3 ± 0.3 ^d^	16.3 ± 0.3 ^a^
H10	14.5 ± 0.5 ^bc^	14.5 ± 0.5 ^cd^	15.1 ± 0.1 ^b^
H20	12.8 ± 0.8 ^b^	12.8 ± 0.3 ^bc^	14.0 ± 1.0 ^ab^
H40	10.3 ± 0.3 ^ba^	11.5 ± 0.5 ^ab^	12.5 ± 0.5 ^ab^
H60	9.0 ± 0.0 ^a^	9.8 ± 0.3 ^a^	11.0 ± 0.0 ^a^

^(1)^ Values (Mean ± SD) with the same letter within the same column are not significantly different (*p* > 0.05) according to Tukey’s HSD test. L10, L20, L40, and L60 represent WWFs milled at 50 rpm for 10, 20, 40, and 60 min, respectively; H10, H20, H40, and H60 represent WWFs milled at 130 rpm for 10, 20, 40, and 60 min, respectively.

**Table 5:** Total phenolic content and ABTS radical-scavenging activity of the mortar-milled WWF samples.

Sample	Total Phenolic Content (mg GAE/100 g)	ABTS Radical-Scavenging Activity (mg TE/100 g)
L10	426.9 ± 1.8 ^a(1)^	330.6 ± 5.9 ^e^
L20	438.9 ± 1.9 ^b^	326.5 ± 1.1 ^e^
L40	463.4 ± 7.0 ^cd^	274.2 ± 2.2 ^c^
L60	442.7 ± 2.4 ^c^	267.3 ± 5.3 ^bc^
H10	438.8 ± 1.6 ^b^	288.8 ± 3.7 ^d^
H20	460.8 ± 1.1 ^c^	266.1 ± 2.2 ^bc^
H40	467.3 ± 4.8 ^cd^	255.9 ± 2.1 ^a^
H60	472.0 ± 4.1 ^d^	260.5 ± 0.9 ^ab^

^(1)^ Values (Mean ± SD) with the same letter within the same column are not significantly different (*p* > 0.05) according to Tukey’s HSD test. L10, L20, L40, and L60 represent WWFs milled at 50 rpm for 10, 20, 40, and 60 min, respectively; H10, H20, H40, and H60 represent WWFs milled at 130 rpm for 10, 20, 40, and 60 min, respectively.

**Table 6:** Color and texture of fresh noodles prepared from mortar-milled WWFs.

Sample	Water Adjustment	Water Addition (g)	Color			Texture of Fresh Noodles		
			L*	a*	b*	Resistance (N)	Extensibility (mm)	R/E
L10	I	36.7	67.3 ± 1.2 ^fg(1)^	7.6 ± 0.7 ^a^	16.8 ± 1.2 ^a^	0.28 ± 0.02 ^b^	2.41 ± 0.20 ^def^	0.12 ± 0.01 ^a^
	II	38.0	63.1 ± 0.6 ^cd^	9.3 ± 0.3 ^b^	19.4 ± 0.8 ^b^	0.34 ± 0.02 ^bcd^	2.58 ± 0.05 ^fgh^	0.13 ± 0.01 ^ab^
L20	I	37.1	64.9 ± 0.7 ^de^	8.2 ± 0.3 ^a^	17.7 ± 0.5 ^a^	0.33 ± 0.04 ^bc^	2.46 ± 0.19 ^def^	0.14 ± 0.02 ^ab^
	II	39.2	61.7 ± 0.2 ^bc^	9.3 ± 0.1 ^b^	19.4 ± 0.4 ^b^	0.36 ± 0.03 ^cde^	2.49 ± 0.05 ^efg^	0.15 ± 0.01 ^b^
L40	I	37.6	66.0 ± 0.1 ^ef^	7.9 ± 0.1 ^a^	16.8 ± 0.4 ^a^	0.34 ± 0.0 ^cd^	2.44 ± 0.03 ^def^	0.14 ± 0.01 ^ab^
	II	41.0	60.6 ± 0.2 ^ab^	9.8 ± 0.2 ^bc^	20.0 ± 0.3 ^b^	0.40 ± 0.03 ^e^	2.71 ± 0.05 ^ghi^	0.15 ± 0.01 ^b^
L60	I	38.2	65.9 ± 0.4 ^ef^	8.1 ± 0.3 ^a^	17.2 ± 0.6 ^a^	0.38 ± 0.03 ^cde^	2.55 ± 0.06 ^cd^	0.15 ± 0.01 ^b^
	II	42.6	61.4 ± 0.3 ^bc^	9.3 ± 0.1 ^b^	19.4 ± 0.1 ^b^	0.38 ± 0.03 ^cde^	2.70 ± 0.02 ^fgh^	0.14 ± 0.01 ^ab^
H10	I	37.9	65.0 ± 1.7 ^de^	8.1 ± 0.4 ^a^	17.2 ± 0.8 ^a^	0.39 ± 0.03 ^de^	2.78 ± 0.16 ^hi^	0.14 ± 0.01 ^ab^
	II	41.9	61.2 ± 0.1 ^bc^	9.9 ± 0.1 ^bc^	20.5 ± 0.1 ^b^	0.39 ± 0.02 ^de^	2.87 ± 0.04 ^i^	0.14 ± 0.01 ^ab^
H20	I	39.3	66.2 ± 0.3 ^efg^	7.8 ± 0.1 ^a^	16.5 ± 0.3 ^a^	0.36 ± 0.03 ^cde^	2.44 ± 0.10 ^def^	0.15 ± 0.01 ^b^
	II	46.2	59.8 ± 0.1 ^ab^	10.3 ± 0.1 ^c^	20.9 ± 0.1 ^b^	0.40 ± 0.02 ^e^	2.23 ± 0.06 ^d^	0.18 ± 0.01 ^c^
H40	I	41.7	68.1 ± 0.6 ^g^	7.4 ± 0.5 ^a^	16.2 ± 0.9 ^a^	0.33 ± 0.01 ^bc^	2.28 ± 0.16 ^de^	0.15 ± 0.01 ^b^
	II	53.5	60.8 ± 0.6 ^ab^	9.6 ± 0.3 ^bc^	19.8 ± 0.2 ^b^	0.18 ± 0.01 ^a^	1.53 ± 0.10 ^b^	0.11 ± 0.01 ^a^
H60	I	44.5	66.1 ± 1.3 ^efg^	7.8 ± 0.3 ^a^	16.6 ± 0.5 ^a^	0.33 ± 0.02 ^bc^	1.78 ± 0.07 ^c^	0.19 ± 0.01 ^c^
	II	61.9	59.1 ± 0.2 ^a^	9.7 ± 0.2 ^bc^	19.7 ± 0.2 ^b^	0.13 ± 0.01 ^a^	0.97 ± 0.10 ^a^	0.13 ± 0.02 ^ab^

^(1)^ Values (Mean ± SD) with the different lowercase letters within the same column are significantly different (*p* < 0.05) according to Tukey’s HSD test. L10, L20, L40, and L60 represent WWFs milled at 50 rpm for 10, 20, 40, and 60 min, respectively; H10, H20, H40, and H60 represent WWFs milled at 130 rpm for 10, 20, 40, and 60 min, respectively.

**Table 7:** Weight gain, turbidity of cooking water, and textural characteristics of cooked noodles made from mortar-milled WWFs.

Sample	Water Adjustment	Weight Gain (%)	Turbidity (ΔA hr^−1^ g flour^−1^)	Textural Parameter of Cooked Noodles				
				Firmness (N)	Resilience	Cohesiveness	Springiness	Chewiness (mJ)
L10	I	252 ± 8 ^bc(1)^	1.20 ± 0.01 ^j^	5.8 ± 0.2 ^b^	0.12 ± 0.01 ^ab^	0.34 ± 0.02 ^abc^	0.65 ± 0.04 ^a^	2.2 ± 0.2 ^b^
	II	259 ± 9 ^bc^	1.41 ± 0.01 ^k^	5.5 ± 0.4 ^b^	0.11 ± 0.02 ^a^	0.31 ± 0.03 ^a^	0.64 ± 0.04 ^a^	2.0 ± 0.4 ^b^
L20	I	255 ± 0 ^bc^	1.12 ± 0.01 ^gh^	5.9 ± 0.2 ^b^	0.13 ± 0.01 ^abc^	0.35 ± 0.02 ^abcd^	0.67 ± 0.05 ^abc^	2.4 ± 0.2 ^bc^
	II	254 ± 0 ^bc^	0.91 ± 0.01 ^b^	6.4 ± 0.3 ^c^	0.11 ± 0.01 ^ab^	0.33 ± 0.03 ^ab^	0.67 ± 0.04 ^ab^	2.4 ± 0.3 ^bc^
L40	I	241 ± 1 ^abc^	1.14 ± 0.02 ^h^	6.9 ± 0.3 ^de^	0.12 ± 0.02 ^ab^	0.35 ± 0.02 ^abcd^	0.68 ± 0.02 ^abcd^	2.8 ± 0.3 ^cd^
	II	248 ± 2 ^bc^	0.94 ± 0.00 ^c^	7.0 ± 0.4 ^def^	0.14 ± 0.01 ^bcd^	0.38 ± 0.03 ^cdef^	0.72 ± 0.03 ^cdef^	3.1 ± 0.3 ^de^
L60	I	236 ± 3 ^ab^	1.01 ± 0.00 ^d^	6.7 ± 0.3 ^cde^	0.13 ± 0.02 ^abc^	0.35 ± 0.02 ^abcd^	0.68 ± 0.04 ^abcd^	2.9 ± 0.3 ^cd^
	II	247 ± 1 ^bc^	1.16 ± 0.00 ^i^	7.3 ± 0.2 ^fg^	0.13 ± 0.02 ^bcd^	0.39 ± 0.03 ^def^	0.74 ± 0.03 ^ef^	3.5 ± 0.4 ^e^
H10	I	268 ± 8 ^c^	1.12 ± 0.01 ^g^	6.9 ± 0.2 ^de^	0.13 ± 0.02 ^abc^	0.36 ± 0.04 ^bcde^	0.68 ± 0.03 ^abcd^	2.9 ± 0.4 ^cd^
	II	248 ± 7 ^bc^	0.77 ± 0.00 ^a^	7.3 ± 0.3 ^fg^	0.13 ± 0.02 ^bcd^	0.37 ± 0.03 ^cdef^	0.71 ± 0.03 ^bcdef^	3.1 ± 0.3 ^de^
H20	I	260 ± 6 ^bc^	1.03 ± 0.01 ^e^	7.1 ± 0.3 ^ef^	0.13 ± 0.02 ^bcd^	0.38 ± 0.03 ^cdef^	0.70 ± 0.03 ^bcde^	3.2 ± 0.4 ^de^
	II	240 ± 6 ^abc^	1.21 ± 0.01 ^j^	6.7 ± 0.2 ^cd^	0.15 ± 0.01 ^cd^	0.40 ± 0.01 ^ef^	0.74 ± 0.03 ^ef^	3.0 ± 0.1 ^de^
H40	I	251 ± 7 ^bc^	1.07 ± 0.01 ^f^	8.2 ± 0.1 ^h^	0.16 ± 0.01 ^de^	0.41 ± 0.02 ^fg^	0.73 ± 0.03 ^def^	4.2 ± 0.4 ^f^
	II	213 ± 7 ^a^	1.91 ± 0.01 ^l^	3.2 ± 0.3 ^a^	0.11 ± 0.02 ^ab^	0.37 ± 0.02 ^bcdef^	0.72 ± 0.02 ^bcdef^	1.1 ± 0.0 ^a^
H60	I	256 ± 15 ^bc^	1.08 ± 0.00 ^f^	7.6 ± 0.1 ^g^	0.18 ± 0.02 ^e^	0.45 ± 0.03 ^g^	0.76 ± 0.03 ^f^	4.2 ± 0.4 ^f^
	II	211 ± 10 ^a^	2.21 ± 0.02 ^m^	N/A				

^(1)^ Values (Mean ± SD) with the different lowercase letters within the same column are significantly different (*p* < 0.05) according to Tukey’s HSD test. L10, L20, L40, and L60 represent WWFs milled at 50 rpm for 10, 20, 40, and 60 min, respectively; H10, H20, H40, and H60 represent WWFs milled at 130 rpm for 10, 20, 40, and 60 min, respectively. N/A, not applicable.

## Data Availability

The original contributions presented in this study are included in the article. Further inquiries can be directed to the corresponding author.

## Abbreviations

The following abbreviations are used in this manuscript:

WWF	Whole-wheat flour
SRC	Solvent-retention capacity
SDS	Sodium dodecyl sulfate
TPC	Total phenolic content
TPA	Texture profile analysis
RVA	Rapid viscosity analyzer
ABTS	2,2′-aznio-bis(3-ethylbenzothiazoline-6-sulfonic acid)
R/E	Resistance to extensibility ratio
AACC	American Association of Cereal Chemists
